# Efficacy of capecitabine in patients with locally advanced or metastatic breast cancer with or without prior treatment with fluoropyrimidine: a retrospective study

**DOI:** 10.1007/s00280-018-3617-5

**Published:** 2018-06-05

**Authors:** Sakura Iizumi, Akihiko Shimomura, Tatsunori Shimoi, Kazuki Sudo, Emi Noguchi, Kan Yonemori, Chikako Shimizu, Yasuhiro Fujiwara, Kenji Tamura

**Affiliations:** 1 Department of Breast and Medical Oncology, National Cancer Center Hospital, 5-1-1 Tsukiji, Chuo-ku, Tokyo, 104-0045 Japan; 2 Keio University Graduate School of Medicine, 35 Shinanomachi, Shinjuku-ku, Tokyo, 160-8582 Japan

**Keywords:** Breast neoplasms, Capecitabine, Efficacy, Safety, Fluoropyrimidine

## Abstract

**Purpose:**

We conducted a retrospective study to assess the outcomes of capecitabine for advanced breast cancer (ABC) after perioperative fluoropyrimidines (FPs).

**Methods:**

The charts of patients with ABC who received capecitabine between 2008 and 2016 at the National Cancer Center Hospital (Tokyo, Japan) were reviewed. Progression-free survival (PFS), overall survival (OS), tumor response, and adverse events (AEs) were compared between two groups: an FP group (prior perioperative FP use) and a non-FP group (no prior FP use).

**Results:**

Overall, 288 patients (FP *n* = 105; non-FP *n* = 183) were analyzed. The two groups had similar patient characteristics. The FP group had significantly poorer PFS than the non-FP group (multivariate hazard ratio [HR] 1.33; 95% confidence interval [CI] 1.02–1.73; *p* = 0.036), although the OS did not differ significantly between the groups (multivariate HR 1.00; 95% CI 0.67–1.50; *p* = 0.994). With different cut-off values (relapse-free interval [RFI] = 3, 4, and 5 years), multivariate HRs for PFS were 1.32–1.67 (short RFI), and 1.00–1.25 (long RFI). A trend for a larger HR in the FP group compared to the non-FP group with short RFI than in that with long RFI was also seen for OS. Response rate (RR) and disease control rate (DCR) did not differ significantly between the groups (RR in FP vs non-FP 13.8 vs 21.0%; *p* = 0.173; DCR 54.0 vs 59.9%; *p* = 0.418). No significant difference in AEs existed between the groups.

**Conclusions:**

Extra caution is needed when capecitabine is considered for patients with ABC who used perioperative FP, especially those who had early recurrence.

**Electronic supplementary material:**

The online version of this article (10.1007/s00280-018-3617-5) contains supplementary material, which is available to authorized users.

## Introduction

Breast cancer is the most common cause of death from cancer in female patients [1]. Although the early breast cancer is curable with resection with or without neoadjuvant or adjuvant treatment, advanced breast cancer (ABC) remains incurable. Chemotherapy is one of the important treatment options to achieve the goal for ABC, which is to prolong survival and to maintain quality of life. While anthracyclines and taxanes are used for the first-line chemotherapy for ABC, in addition to their usage in neoadjuvant and adjuvant settings, no single standard regimen exists after failure of these agents [2].

Capecitabine is an orally available fluoropyrimidine (FP), and a prodrug of 5-fluorouracil (5-FU). It is designed to deliver 5-FU preferentially to tumor tissue to enhance efficacy, and reduce toxicities in the gastrointestinal tract and bone marrow. Its route of administration and its favorable toxicity profile, with little alopecia and neuropathy, make it an attractive option for some patients. Phase II trials have revealed the efficacy and safety of capecitabine for ABC after failure of anthracyclines and taxanes [3–6]. Moreover, a phase III trial has shown the benefit of capecitabine in combination with lapatinib in human epidermal growth factor receptor 2 [HER2]-positive patients [7]. Capecitabine is also often used as a comparator in phase III trials [8, 9].

In addition to capecitabine, other FPs such as 5-FU, tegafur-uracil (UFT), and doxifluridine have been used in neoadjuvant and adjuvant therapies for breast cancer. As such, some patients with ABC receiving capecitabine have a previous history of treatment with other FPs. Re-challenge of chemotherapeutic agents has been assessed in other cancers [10–12]. However, aside from a few studies providing limited results, separately assessing patients either with or without prior use of FPs [4, 13], the efficacy of capecitabine in patients with ABC who have received FPs for the early breast cancer has not been studied sufficiently.

Therefore, we conducted a retrospective study to assess the efficacy and safety of capecitabine in patients with ABC with or without prior treatment with FPs.

## Patients and methods

### Patients

We reviewed the medical records of patients with pathologically confirmed locally advanced or metastatic breast cancer, with the previous treatment with surgery of curative intent. All patients had undergone palliative chemotherapy with capecitabine as monotherapy between July 2008 and December 2016 at the National Cancer Center Hospital (Tokyo, Japan). Patients who had received FP-containing regimens as neoadjuvant or adjuvant therapy before capecitabine were assigned to the FP group; patients who had never received FPs were assigned to the non-FP group. Patients who received more than one FP before capecitabine, who received FPs for purposes other than perioperative (neoadjuvant or adjuvant) therapy, and those with insufficient baseline data were excluded from the analysis. This study was approved by the National Cancer Center Institutional Review Board (No. 2016-491). Because this study was retrospective in nature, written informed consent was not obtained. This study was publicized via the web page of the hospital.

### Treatment

Patients received oral capecitabine by one of the following dosing regimens: (1) 1250 mg/m^2^ twice daily for 14 days, followed by a 7-day rest period in a 21-day cycle, (2) 850 mg/m^2^ twice daily for 21 days, followed by a 7-day rest period in a 28-day cycle. The dosing was adjusted according to modifications recommended by the FDA [14], by adverse events, or as per the physicians’ judgment. Treatment cycles were repeated until disease progression or unacceptable toxicity, or until the patients’ wish to terminate treatment.

### Assessment

Tumor response was assessed according to the Response Evaluation Criteria in Solid Tumors, version 1.1 [15] by computed tomography scans. Confirmation of response was not required. Response rate (RR) was defined as the proportion of patients who achieved complete or partial response, while disease control rate (DCR) was defined as the proportion of patients who achieved complete, partial, or stable disease as best response. Progression-free survival (PFS) was defined as the time from the initiation of capecitabine monotherapy, until either clinical or objective disease progression, or death. Overall survival (OS) was defined as the time from the initiation of capecitabine monotherapy until death. Relapse-free interval (RFI) was defined as the time from definitive surgery for breast cancer until recurrence. Adverse events (AEs) were assessed according to the Common Terminology Criteria for Adverse Events, version 4.0.

### Statistical analysis

The study was designed to compare the efficacy (RR, DCR, PFS, and OS) and safety (frequency of grade 3 or worse AEs, AEs requiring hospitalization, and discontinuation due to AEs) of capecitabine between the FP group and the non-FP group. Nominal variables and continuous variables were compared by Fisher’s exact test and the Mann–Whitney *U* test, respectively. Only patients with target lesions were analyzed for RR and DCR. Survival curves were obtained by the Kaplan–Meier method, and differences between the two groups were assessed by the log-rank test. Hazard ratios (HRs) and confidence intervals (CIs) were estimated by the Cox proportional hazards model. Baseline characteristics with *p* value < 0.10 in univariate analysis were adjusted for in multivariate analysis. HRs were adjusted for additional baseline characteristics in various multivariate analysis models. HRs were also estimated for subgroups by biomarkers: the triple-negative subgroup, the hormone-positive subgroup, and the hormone-negative subgroup. To assess the impact of prior use of FPs on survival outcomes by RFI, HRs for PFS and OS were also estimated separately for short RFI and long RFI. Continuous variables were divided into two groups at median. Tests were considered significant if the two-sided *p* value was < 0.05. Analyses were performed with EZR software (Saitama Medical Center, Jichi Medical University, Saitama, Japan), which is a graphical user interface for R (The R foundation for Statistical Computing, Vienna, Austria) [16].

## Results

### Patients

Overall, 288 patients were included in the analysis: 105 were included in the FP group and 183 in the non-FP group (Supplementary Fig. 1). Baseline patient characteristics did not differ significantly between the two groups (Table 1). The median age was 60 (range 25–84) years in the FP group and 59 (range 32–81) years in the non-FP group (*p* = 0.361). The median number of the previous lines of chemotherapy for advanced disease was 1 (range 0–4; *p* = 0.182) in both groups. The median RFI was 3.85 (range 0.27–20.11) years in the FP group and 4.24 (range 0.27–27.07) years in the non-FP group (*p* = 0.369). In the FP group, 86 (81.9%), 8 (7.6%), and 11 (10.5%) patients had received 5-FU, UFT, and doxifluridine, respectively, for a neoadjuvant or adjuvant therapy. The initial diagnosis (preoperative, clinical) was stages I, II, and III in 10 (13.0%), 45 (58.4%), and 22 (28.6%) patients in the FP group, respectively, and 16 (10.5%), 107 (70.4%), and 29 (19.1%) patients in the non-FP group, respectively (*p* = 0.175). The median follow-up time was 12.5 (range 0.1–95.6) months.

**Table 1:** Patient characteristics

	FP	Non-FP	*p* value
*n*	105	183	
Age			
Median [range]	60 [25–84]	59 [32–81]	0.361
ECOG PS			
0 (%)	46 (43.8)	85 (46.4)	0.246
1 (%)	50 (47.6)	91 (49.7)	
2 (%)	9 (8.6)	7 (3.8)	
Biomarker			
ER (%)	84 (80.0)	149 (81.4)	0.758
PgR (%)	76 (72.4)	129 (70.5)	0.788
HER2 (%)	6 (5.8)	6 (3.3)	0.365
Organs involved			
Bone (%)	64 (61.0)	102 (55.7)	0.457
Liver (%)	53 (50.5)	105 (57.4)	0.270
Lymph node (%)	46 (43.8)	84 (45.9)	0.806
Lung (%)	46 (43.8)	79 (43.2)	1.000
No. of organs involved			
Median [range]	2 [1–5]	2 [1–7]	0.067
Previous treatment			
Endocrine therapy (%)	88 (83.8)	151 (82.5)	0.871
Anthracycline (%)	91 (86.7)	154 (84.2)	0.610
Taxane (%)	100 (95.2)	168 (91.8)	0.340
No. of Cx lines^a^			
Median [range]	1 [0–4]	1 [0–4]	0.182
RFI (years)			
Median [range]	3.85 [0.27–20.11]	4.24 [0.27–27.07]	0.369
Stage^b^			
I	10 (13.0)	16 (10.5)	0.175
II	45 (58.4)	107 (70.4)	
III	22 (28.6)	29 (19.1)	
FP type			
5-FU	86 (81.9)	–	
UFT	8 (7.6)	–	
Doxifluridine	11 (10.5)	–	
Capecitabine schedule			
21 days/cycle	63 (60.0)	98 (53.6)	0.325
28 days/cycle	42 (40.0)	85 (46.4)	

*FP* fluoropyrimidine, *ECOG* Eastern Cooperative Oncology Group, *PS* performance status, *ER* estrogen receptor, *PgR* progesterone receptor, *HER2* human epidermal growth factor receptor 2, *Cx* chemotherapy, *RFI* relapse-free interval, *5-FU* 5-fluorouracil, *UFT* tegafur/uracil

^a^For advanced disease

^b^Preoperative clinical stage

### Progression-free survival and overall survival

**Fig. 1:**
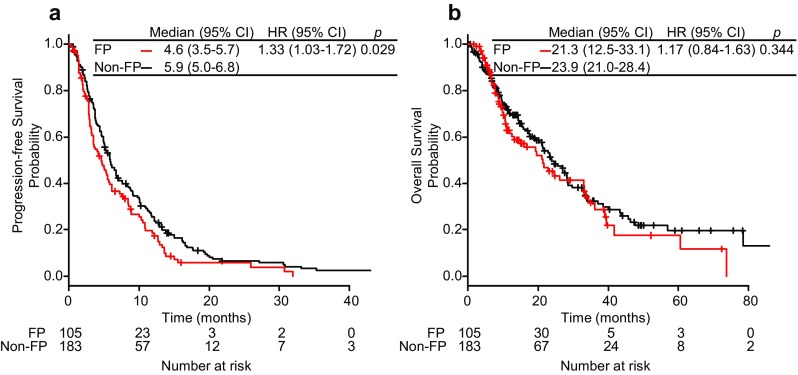
Kaplan–Meier curves of **a** progression-free survival, **b** overall survival. *FP* fluoropyrimidine, *HR* hazard ratio, *CI* confidence interval

The PFS period was significantly shorter in the FP group than in the non-FP group (median 4.6 vs 5.9 months; HR for the FP group compared with the non-FP group: 1.33; 95% CI 1.03–1.72, *p* = 0.029; Fig. 1). In multivariate analysis adjusting for baseline characteristics selected by univariate analysis (age, Eastern Cooperative Oncology Group [ECOG] performance status [PS], estrogen receptor [ER], number [No.] of organs involved, previous endocrine therapy, previous taxane use, and RFI), PFS was worse in the FP group than in the non-FP group (HR 1.33; 95% CI 1.02–1.73; *p* = 0.036; Table 2). The median OS was 21.3 months in the FP group and 23.9 months in the non-FP group (HR 1.17; 95% CI 0.84–1.63; *p* = 0.344). The HR adjusted for baseline characteristics selected by univariate analysis (age, ECOG PS, ER, HER2, bone metastasis, liver metastasis, No. of organs involved, previous therapy [endocrine, anthracycline, and taxane], RFI, and preoperative stage) was 1.00 (95% CI 0.67–1.50; *p* = 0.994; Table 3). Of note, preoperative stage was not selected by univariate analysis for multivariate analysis of PFS; it was selected for multivariate analysis of OS.

**Table 2:** Univariate and multivariate analyses of progression**-**free survival

	Univariate		Multivariate	
	HR (95% CI)	*p* value	HR (95% CI)	*p* value
Age				
≥ 60 vs < 60	0.72 (0.56–0.92)	0.009	0.76 (0.59–0.99)	0.046
ECOG PS				
1 vs 0	1.28 (1.00–1.65)	0.052	1.19 (0.92–1.54)	0.184
2 vs 0	1.89 (1.06–3.36)	0.031	1.60 (0.89–2.88)	0.116
ER				
+ vs −	0.64 (0.47–0.87)	0.004	0.94 (0.58–1.53)	0.813
PgR				
+ vs −	1.04 (0.79–1.37)	0.773		
HER2				
+ vs −	1.48 (0.81–2.72)	0.204		
Bone metastasis				
+ vs −	1.07 (0.83–1.38)	0.594		
Liver metastasis				
+ vs −	1.14 (0.89–1.46)	0.300		
Lymph node metastasis				
+ vs −	1.20 (0.94–1.54)	0.142		
Lung metastasis				
+ vs −	1.15 (0.89–1.47)	0.281		
No. of metastatic organs				
≥ 2 vs < 2	1.39 (1.00–1.94)	0.050	1.47 (1.02–2.11)	0.037
Endocrine therapy				
+ vs −	0.62 (0.45–0.86)	0.004	0.59 (0.35–0.98)	0.042
Anthracycline				
+ vs −	1.33 (0.93–1.88)	0.115		
Taxane				
+ vs −	1.59 (0.94–2.69)	0.081	1.25 (0.73–2.14)	0.412
No. of Cx lines^a^				
≥ 1 vs < 1	1.09 (0.82–1.43)	0.563		
FP				
FP vs non-FP	1.33 (1.03–1.72)	0.030	1.33 (1.02–1.73)	0.036
RFI (years)				
Continuous (per year)	0.99 (0.96–1.01)	0.299		
≥ 4 vs < 4	0.73 (0.57–0.93)	0.012	0.85 (0.64–1.11)	0.229
Stage^b^				
II vs I	1.05 (0.67–1.63)	0.833		
III vs I	0.85 (0.51–1.41)	0.527		
Capecitabine schedule				
28 vs 21 days	1.10 (0.86–1.41)	0.464		

Cox proportional hazards model. Covariates with *p* value < 0.10 were adjusted in multivariate analysis

*HR* hazard ratio, *CI* confidence interval, *ECOG* Eastern Cooperative Oncology Group, *PS* performance status, *ER* estrogen receptor, *PgR* progesterone receptor, *HER2* human epidermal growth factor receptor 2, *Cx* chemotherapy, *FP* fluoropyrimidine, *RFI* relapse-free survival

^a^For advanced disease

^b^Preoperative clinical stage

**Table 3:** Univariate and multivariate analyses of overall survival

	Univariate		Multivariate	
	HR (95% CI)	*p* value	HR (95% CI)	*p* value
Age				
≥ 60 vs < 60	0.71 (0.52–0.98)	0.037	0.95 (0.65–1.40)	0.802
ECOG PS				
1 vs 0	2.01 (1.44–2.81)	< 0.001	2.22 (1.51–3.27)	< 0.001
2 vs 0	4.96 (2.57–9.57)	< 0.001	6.89 (3.11–15.29)	< 0.001
ER				
+ vs −	0.62 (0.43–0.89)	0.011	0.35 (0.16–0.75)	0.007
PgR				
+ vs −	0.93 (0.66–1.31)	0.673		
HER2				
+ vs −	1.93 (0.94–3.95)	0.073	3.82 (1.53–9.52)	0.004
Bone metastasis				
+ vs −	1.39 (1.00–1.92)	0.047	1.23 (0.79–1.93)	0.365
Liver metastasis				
+ vs −	1.62 (1.18–2.23)	0.003	1.75 (1.12–2.75)	0.014
Lymph node metastasis				
+ vs −	1.30 (0.95–1.79)	0.100		
Lung metastasis				
+ vs −	0.85 (0.61–1.17)	0.314		
No. of metastatic organs				
≥ 2 vs < 2	1.73 (1.11–2.69)	0.015	1.29 (0.72–2.32)	0.385
Endocrine therapy				
+ vs −	0.61 (0.41–0.91)	0.016	1.15 (0.53–2.48)	0.722
Anthracycline				
+ vs −	1.49 (0.93–2.38)	0.098	0.78 (0.42–1.46)	0.441
Taxane				
+ vs −	1.92 (0.90–4.11)	0.093	1.37 (0.45–4.19)	0.580
No. of Cx lines^a^				
≥ 1 vs < 1	0.93 (0.66–1.31)	0.687		
FP				
FP vs non-FP	1.17 (0.84–1.63)	0.345	1.00 (0.67–1.50)	0.994
RFI (years)				
Continuous (per year)	0.97 (0.94–1.01)	0.123		
≥ 4 vs < 4	0.71 (0.52–0.98)	0.035	0.98 (0.66–1.46)	0.914
Stage^b^				
II vs I	1.67 (0.93–3.00)	0.084	1.57 (0.84–2.92)	0.160
III vs I	1.21 (0.62–2.37)	0.570	1.05 (0.51–2.16)	0.901
Capecitabine schedule				
28 vs 21 days	1.08 (0.79–1.49)	0.634		

Cox proportional hazards model. Covariates with *p* value < 0.10 were adjusted in multivariate analysis

*HR* hazard ratio, *CI* confidence interval, *ECOG* Eastern Cooperative Oncology Group, *PS* performance status, *ER* estrogen receptor, *PgR* progesterone receptor, *HER2* human epidermal growth factor receptor 2, *Cx* chemotherapy, *FP* fluoropyrimidine, *RFI* relapse-free survival

^a^For advanced disease

^b^Preoperative clinical stage

The HRs for PFS and OS largely did not change in additional multivariate models further adjusting for different baseline characteristics: liver and lung metastasis, all biomarker characteristics, all previous treatment characteristics, and age ≥ 35 vs < 35 instead of ≥ 60 vs < 60 (considering that age < 60 was associated with worse PFS and OS than was age < 60 in univariate analysis, and that age ≥ 35 has been reported to be a negative prognostic factor for the early breast cancer, although its prognostic effect is still unclear in an advanced disease setting [17]) (Supplementary Table 1). Prior FP use was not linked to PFS and OS, in the analysis performed according to biomarker status (Supplementary Table 2).

The HRs for PFS and OS in the FP group compared with the non-FP group were also estimated separately for short RFI and long RFI, with a cutoff at 4 years (median RFI) (Table 4). The multivariate-adjusted HRs for PFS were 1.56 (95% CI 1.06–2.28; *p* = 0.025) with short RFI, and 1.11 (95% CI 0.76–1.60; *p* = 0.597) with long RFI. The multivariate-adjusted HRs for OS were 1.23 (95% CI 0.68–2.21; *p* = 0.489) with short RFI, and 0.77 (95% CI 0.40–1.47; *p* = 0.427) with long RFI. A trend for larger HRs for PFS and OS in the FP group with short RFI than in that with long RFI was also observed with different cutoffs of RFI (Supplementary Table 3).

**Table 4:** Univariate and multivariate analyses of progression**-**free and overall survival for the FP group compared with the non-FP group by relapse-free survival

RFI (years)	Univariate		Multivariate^a^	
	HR (95% CI)	*p* value	HR (95% CI)	*p* value
PFS				
< 4	1.49 (1.02–2.18)	0.037	1.56 (1.06–2.28)	0.025
≥ 4	1.19 (0.83–1.70)	0.344	1.11 (0.76–1.60)	0.597
OS				
< 4	1.28 (0.81–2.04)	0.288	1.23 (0.68–2.21)	0.489
≥ 4	1.01 (0.63–1.62)	0.959	0.77 (0.40–1.47)	0.427

Cox proportional hazards model

*FP* fluoropyrimidine, *RFI* relapse-free survival, *HR* hazard ratio, *CI* confidence interval, *PFS* progression-free survival, *OS* overall survival

^a^Adjusting for covariates with *p* value < 0.10 in univariate analysis except for RFI < 4 vs ≥ 4 years

### Tumor response

Tumor response among patients with target lesions is shown in Supplementary Table 4. The RR (FP vs non-FP) was 13.8 vs 21.0% (*p* = 0.173) and DCR was 54.0 vs 59.9% (*p* = 0.418).

### Safety

Table 5 shows the frequency of AEs by group. There was no significant difference between the two groups in grade 3 or worse AEs, AEs requiring hospitalization, or treatment discontinuation due to AEs. Hand–foot syndrome was the most frequent cause of discontinuation due to AEs in both groups (2.9 vs 2.7%). There were no treatment-related deaths. The frequencies of dose interruptions and reductions did not differ significantly between the two groups (dose interruptions 44.8 vs 53.0%, *p* = 0.221; dose reductions 39.0 vs 49.7%, *p* = 0.087).

**Table 5:** Adverse events of Grade 3 or higher

	FP	Non-FP	*p* value
*n*	105	183	
All (%)	27 (25.7)	51 (27.9)	0.783
Hematological (%)	17 (16.2)	30 (16.4)	1.000
Leukopenia (%)	2 (1.9)	12 (6.6)	0.092
Neutropenia (%)	9 (8.6)	15 (8.2)	1.000
Anemia (%)	4 (3.8)	9 (4.9)	0.775
Thrombocytopenia (%)	4 (3.8)	5 (2.7)	0.728
Febrile neutropenia (%)	1 (1.0)	2 (1.1)	1.000
Nonhematological (%)	16 (15.2)	31 (16.9)	0.743
Fatigue (%)	0 (0.0)	0 (0.0)	NA
Anorexia (%)	2 (1.9)	1 (0.5)	0.301
Nausea (%)	1 (1.0)	1 (0.5)	1.000
Vomiting (%)	0 (0.0)	1 (0.5)	1.000
Diarrhea (%)	1 (1.0)	2 (1.1)	1.000
Mucositis (%)	0 (0.0)	1 (0.5)	1.000
HFS (%)	6 (5.7)	5 (2.7)	0.217
Bilirubin increased (%)	1 (1.0)	2 (1.1)	1.000
AST increased (%)	7 (6.7)	14 (7.7)	0.818
ALT increased (%)	8 (7.6)	15 (8.2)	1.000
Creatinine increased (%)	0 (0.0)	0 (0.0)	NA
Other (%)	1^a^ (1.0)	2^b^ (1.1)	1.000
Hospitalization (%)	3^c^ (2.9)	4^d^ (2.2)	0.708
Discontinuation (%)	3^e^ (2.9)	6^f^ (3.3)	1.000

*FP* fluoropyrimidines, *HFS* hand–foot syndrome, *AST* aspartate aminotransferase, *ALT* alanine aminotransferase, *NA* not assessed

^a^Lung infection grade 3

^b^Lung infection grade 3 and enterocolitis infectious grade 3

^c^Lung infection grade 3 and anorexia grade 3 (2 patients)

^d^Lung infection grade 3, enterocolitis infectious grade 3, diarrhea grade 3, and mucositis grade 3

^e^Hand–foot syndrome grade 3 (2 patient), hand–foot syndrome grade 2

^f^Hand–foot syndrome grade 3 (2 patient), hand–foot syndrome grade 2 (3 patients), diarrhea grade 3

## Discussion

In this study, we retrospectively evaluated the efficacy and safety of capecitabine monotherapy for the treatment of ABC in patients who had received other FPs for neoadjuvant or adjuvant therapy. Although OS did not differ significantly with prior FP use, PFS was worse in patients with prior FP use. The detrimental effect of prior FP use on survival outcomes seemed larger in patients with the early recurrence after surgery than in those with late recurrence. The safety profile did not differ significantly by prior FP use.

In addition to its use in ABC, the efficacy of capecitabine has been explored in the early breast cancer treatment. A recent meta-analysis of randomized-controlled studies demonstrated that addition of capecitabine to standard chemotherapy improves survival outcomes in the early breast cancer, particularly in triple-negative breast cancer [18]. In addition to the clinical trial CREATE-X, which showed the efficacy of adjuvant capecitabine in patients with residual disease after neoadjuvant chemotherapy [13], several other trials evaluating adjuvant capecitabine in high-risk patients are ongoing [18]. Such studies may lead to incorporation of capecitabine into the standard perioperative treatment. The current study did not assess patients who had used capecitabine as perioperative FP. Nevertheless, given the efficacy of FPs as perioperative therapies as well as in advanced disease, our results provide insights for clinicians considering treatment options in patients with ABC after perioperative FPs.

Capecitabine may be less effective in patients with prior FP use. In this study, PFS was significantly worse in the FP group, which was confirmed by various multivariate models adjusting for baseline characteristics. In addition, capecitabine appears to be slightly less active after the use of FPs, as shown in subgroup analyses reported in a limited number of the previous studies. In one trial, the response rate of capecitabine for ABC was 13% in patients with prior 5-FU use and 18% (*p* = 0.48) in those without prior 5-FU use [4]. Moreover, in the CREATE-X trial, the HR for disease-free survival with adjuvant capecitabine compared with control was 0.75 (95% CI 0.53–1.05) with prior 5-FU use, and 0.63 (95% CI 0.40–0.99) without prior 5-FU use (*p* = 0.56) [13]. Although capecitabine may remain as a treatment option for ABC pretreated with FPs based on the absence of significant OS differences by prior FP use, we should be aware that it may be less active in ABC patients with prior FP use than in those without prior FP use.

The effect of prior FP use seemed to differ between the early recurrence (short RFI) and late recurrence (long RFI). We used RFI to compare patients with prior FP use and those without prior FP use. The previous studies that assessed re-challenge with a chemotherapeutic agent in other cancers reported that drug-free interval (time since last administration of the drug until relapse) is predictive of the efficacy of re-challenge [10–12]. However, drug-free interval may be dependent upon the nature of the tumor (aggressive or indolent). In addition, these studies did not include a comparison group (patients without prior drug use) and may have compared a group with a better prognosis (long drug-free interval) with a group with poorer prognosis (shorter drug-free interval). In the present study, patients with prior FP use with the early recurrence and those with late recurrence were compared with RFI-matched patients without prior FP use, and the difference in the impact of prior FP use by length of RFI was assessed. Prior FP use in patients with late recurrence appeared to have a less adverse impact on survival outcomes. In contrast, in those with the early recurrence, prior FP use seemed to be detrimental to survival outcomes. This outcome is biologically plausible, since the early recurrence indicates failure of perioperative treatment including FPs, in which case inefficacy of capecitabine could also be suspected.

In the current study, potential differences in preoperative tumor extent were analyzed. It is possible that a higher percentage of patients in the FP group may have had advanced preoperative stage disease necessitating neoadjuvant chemotherapy that contained FPs, compared with that in the non-FP group. It is also possible that preoperative stages may have affected the outcomes of treatment with capecitabine. However, preoperative diagnosis did not have a clear effect on PFS or OS. The HR for PFS seemed to be similar regardless of preoperative diagnosis in the univariate analysis. For OS, while preoperative diagnosis was selected for multivariate analysis, there was no apparent dose–response relationship between preoperative diagnosis and OS in the univariate analysis. Although data of preoperative stage were not available in some patients, we evaluated the effect of difference in preoperative stage on the outcomes, and minimized the potential bias due to such a difference between patients with and without prior preoperative FP use.

There are limitations to this study. First, it remains unclear whether other chemotherapeutic regimens are more efficacious than capecitabine in patients with unfavorable outcomes of capecitabine (those with prior FP use and early recurrence). Studies comparing capecitabine with other agents should be conducted in such patients. Second, it was a retrospective study of a limited sample size and event numbers. For example, perioperative data were difficult to collect, since many patients had surgery in various hospitals, several years prior to capecitabine treatment. In addition, the assessment of PFS and OS by biomarkers was not sufficient for the small number of patients in the subgroups. Furthermore, data on the adherence to treatment were not obtainable from the medical records. Third, the study population was heterogeneous regarding perioperative chemotherapy, which included various regimens.

In conclusion, the use of capecitabine requires extra caution when it is considered for ABC with prior FP use, since PFS seems to be inferior in ABC patients with prior FP use than in those without prior FP use. The RFI may be a factor to consider when clinicians select treatment for patients with prior FP use, as the adverse effect of prior FP use seems to be larger in patients with the early recurrence after surgery, (cases where inefficacy of prior perioperative treatment including FPs is suspected). Whether patients with ABC who received perioperative FP should receive agents other than capecitabine requires further elucidation.

## Electronic supplementary material

Below is the link to the electronic supplementary material.

Supplementary material 1 (PPTX 40 KB)

Supplementary material 2 (DOCX 30 KB)

Supplementary material 3 (DOCX 33 KB)

Supplementary material 4 (DOCX 30 KB)

Supplementary material 5 (DOCX 27 KB)
