# Supplementary material for: Efficacy of capecitabine in patients with locally advanced or metastatic breast cancer with or without prior treatment with fluoropyrimidine: a retrospective study
Source: Cancer Chemother Pharmacol. 2018 Jun 5;82(2):275–83. doi: 10.1007/s00280-018-3617-5 (PMC6060805; doi:10.1007/s00280-018-3617-5)
Supplement: Supplementary file 1 — Supplementary material 1 (PPTX 40 KB) [file 280_2018_3617_MOESM1_ESM.pptx]

## Slide 1
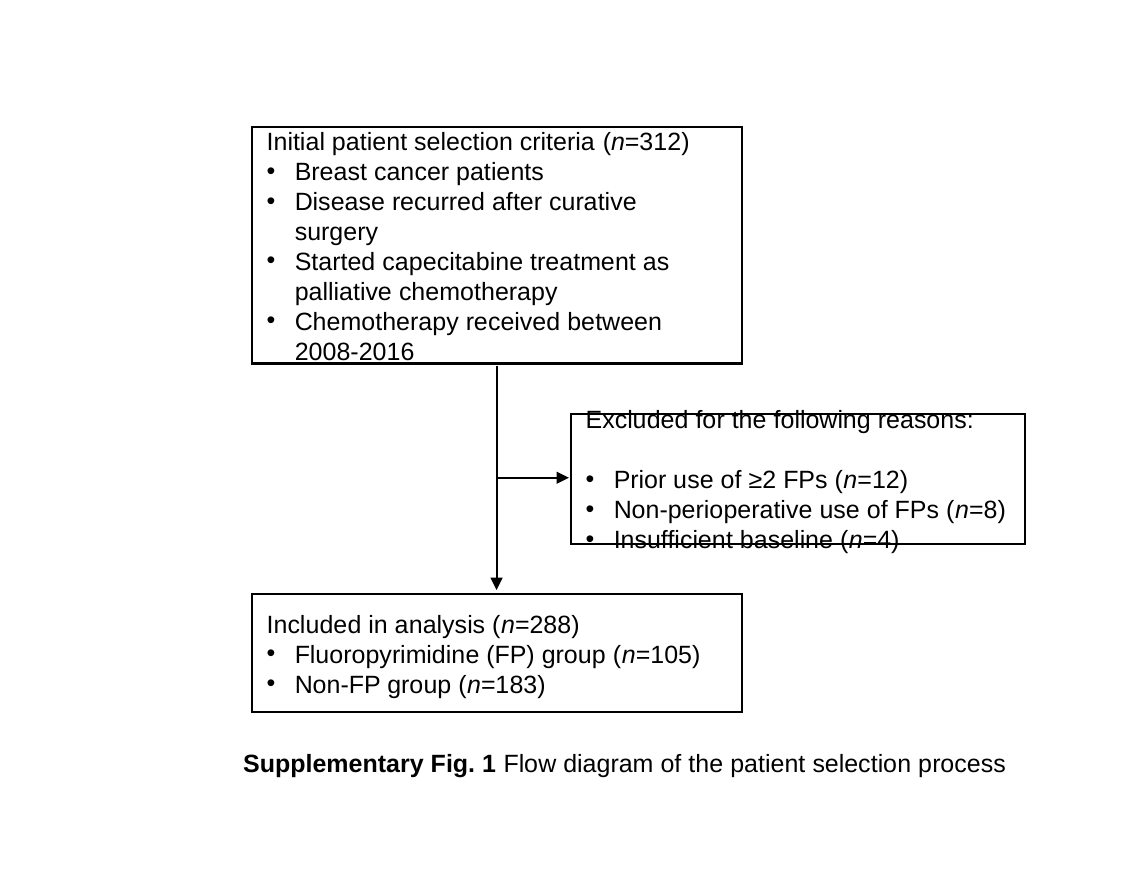

Initial patient selection criteria (n=312)
Breast cancer patients
Disease recurred after curative surgery
Started capecitabine treatment as palliative chemotherapy
Chemotherapy received between 2008-2016
Excluded for the following reasons:
Prior use of ≥2 FPs (n=12)
Non-perioperative use of FPs (n=8)
Insufficient baseline (n=4)
Included in analysis (n=288)
Fluoropyrimidine (FP) group (n=105)
Non-FP group (n=183)
Supplementary Fig. 1 Flow diagram of the patient selection process
