# Supplementary material for: Efficacy of capecitabine in patients with locally advanced or metastatic breast cancer with or without prior treatment with fluoropyrimidine: a retrospective study
Source: Cancer Chemother Pharmacol. 2018 Jun 5;82(2):275–83. doi: 10.1007/s00280-018-3617-5 (PMC6060805; doi:10.1007/s00280-018-3617-5)
Supplement: Supplementary file 2 — Supplementary material 2 (DOCX 30 KB) [file 280_2018_3617_MOESM2_ESM.docx]

**Supplementary Table 1** Hazard ratios for progression-free survival and overall survival further adjusted for different covariates

|  | PFS |  | OS |  |
| --- | --- | --- | --- | --- |
| Model | HR (95% CI) | *p* value | HR (95% CI) | *p* value |
| Visceral organs^a^ | 1.35 (1.03-1.77) | 0.028 | 1.03 (0.68-1.55) | 0.890 |
| All biomarker characteristics^b^ | 1.32 (1.01-1.72) | 0.039 | 1.01 (0.67-1.51) | 0.979 |
| All biomarker characteristics^c^ | 1.31 (1.01-1.71) | 0.044 | 1.05 (0.70-1.57) | 0.833 |
| All previous treatment characteristics^d^ | 1.32 (1.02-1.72) | 0.038 | 1.00 (0.67-1.50) | 0.994 |
| Age　≥35 vs <35^e^ | 1.28 (0.99-1.67) | 0.063 | 0.98 (0.66-1.47) | 0.940 |

Covariates not selected in univariate analysis were further adjusted for. ^a^Covariates with *p*<0.10 in univariate analysis and liver metastasis, and lung metastasis. ^b^Covariates with *p*<0.10 in univariate analysis and all biomarkers (estrogen receptor [ER], progesterone receptor [PgR], and human epidermal growth factor receptor 2 [HER2]). ^c^Covariates with *p*<0.10 in univariate analysis (except for ER), hormone receptor (ER or PgR)-positive/negative, and HER2. ^d^Covariates with *p*<0.10 in univariate analysis and previous treatment (endocrine therapy, taxanes, and anthracyclines). ^e^Covariates with *p*<0.10 in univariate analysis except for age (≥60 vs <60), and age (≥35 vs <35). *PFS* progression-free survival, *OS* overall survival, *HR* hazard ratio, *CI* confidence interval
