# Supplementary material for: Efficacy of capecitabine in patients with locally advanced or metastatic breast cancer with or without prior treatment with fluoropyrimidine: a retrospective study
Source: Cancer Chemother Pharmacol. 2018 Jun 5;82(2):275–83. doi: 10.1007/s00280-018-3617-5 (PMC6060805; doi:10.1007/s00280-018-3617-5)
Supplement: Supplementary file 3 — Supplementary material 3 (DOCX 33 KB) [file 280_2018_3617_MOESM3_ESM.docx]

**Supplementary Table 2** Univariate and multivariate analysis of progression-free and overall survival for different biomarker subgroups

|  |  |  | Univariate |  | Multivariate^a^ |  |
| --- | --- | --- | --- | --- | --- | --- |
|  |  | *n* (FP/non-FP) | HR (95% CI) | *p* value | HR (95% CI) | *p* value |
| PFS | Triple-negative | 16/26 | 1.36 (0.71-2.58) | 0.354 | 1.58 (0.71-3.5) | 0.259 |
|  | Hormone positive^b^ | 88/155 | 1.32 (0.99-1.75) | 0.055 | 1.28 (0.96-1.71) | 0.091 |
|  | Hormone negative^b^ | 17/28 | 1.40 (0.75-2.61) | 0.285 | 1.64 (0.77-3.49) | 0.199 |
|  | HER2+ excluded | 98/175 | 1.29 (0.99-1.68) | 0.061 | 1.29 (0.99-1.69) | 0.061 |
| OS | Triple-negative | 16/26 | 1.69 (0.78-3.62) | 0.181 | 1.28 (0.32-5.16) | 0.731 |
|  | Hormone positive^b^ | 88/155 | 1.09 (0.76-1.58) | 0.636 | 1.00 (0.64-1.56) | 0.994 |
|  | Hormone negative^b^ | 17/28 | 1.60 (0.77-3.33) | 0.211 | 1.58 (0.44-5.65) | 0.479 |
|  | HER2+ excluded^c^ | 98/175 | 1.14 (0.81-1.61) | 0.444 | 1.05 (0.7-1.58) | 0.811 |

Cox proportional hazards model. ^a^Adjusting for covariates with *p* value<0.10 in univariate analysis except for biomarkers and previous endocrine therapy. ^b^Includes both of HER2 positive and negative patients. ^c^Adjusted for covariates with univariate *p* value<0.10 except for HER2 status. *FP* fluoropyrimidine, *HR* hazard ratio, *CI* confidence interval, *PFS* progression-free survival, *HER2* human epidermal growth factor receptor 2, *OS* overall survival
