# Supplementary material for: Efficacy of capecitabine in patients with locally advanced or metastatic breast cancer with or without prior treatment with fluoropyrimidine: a retrospective study
Source: Cancer Chemother Pharmacol. 2018 Jun 5;82(2):275–83. doi: 10.1007/s00280-018-3617-5 (PMC6060805; doi:10.1007/s00280-018-3617-5)
Supplement: Supplementary file 4 — Supplementary material 4 (DOCX 30 KB) [file 280_2018_3617_MOESM4_ESM.docx]

**Supplementary Table 3** Hazard ratios for progression-free survival and overall survival by recurrence-free interval with different cutoffs

|  |  | Univariate |  | Multivariate^a^ |  |
| --- | --- | --- | --- | --- | --- |
|  | RFI (years) | HR (95% CI) | *p* value | HR (95% CI) | *p* value |
| PFS | <3 | 1.34 (0.87-2.06) | 0.191 | 1.32 (0.84-2.06) | 0.224 |
|  | ≥3 | 1.30 (0.94-1.79) | 0.111 | 1.25 (0.89-1.75) | 0.195 |
|  | <5 | 1.57 (1.11-2.22) | 0.011 | 1.67 (1.16-2.39) | 0.005 |
|  | ≥5 | 1.11 (0.75-1.64) | 0.610 | 1.00 (0.66-1.50) | 0.986 |
| OS | <3 | 1.21 (0.72-2.05) | 0.469 | 1.15 (0.59-2.25) | 0.678 |
|  | ≥3 | 1.11 (0.72-1.69) | 0.647 | 0.86 (0.50-1.50) | 0.602 |
|  | <5 | 1.36 (0.89-2.09) | 0.155 | 1.31 (0.76-2.25) | 0.335 |
|  | ≥5 | 0.94 (0.56-1.58) | 0.809 | 0.81 (0.40-1.68) | 0.577 |

^a^Covariates with *p* value<0.10 were adjusted in multivariate analysis except for RFI. *RFI* recurrence-free interval, *HR* hazard ratio, *CI* confidence interval, *PFS* progression-free survival, *OS* overall survival
