# Supplementary material for: Efficacy of capecitabine in patients with locally advanced or metastatic breast cancer with or without prior treatment with fluoropyrimidine: a retrospective study
Source: Cancer Chemother Pharmacol. 2018 Jun 5;82(2):275–83. doi: 10.1007/s00280-018-3617-5 (PMC6060805; doi:10.1007/s00280-018-3617-5)
Supplement: Supplementary file 5 — Supplementary material 5 (DOCX 27 KB) [file 280_2018_3617_MOESM5_ESM.docx]

**Supplementary Table 4** Tumor response

|  |  | FP | Non-FP | *p* value^a^ |
| --- | --- | --- | --- | --- |
| *n* |  | 87 | 157 |  |
| Best response | CR | 1 (1.1) | 0 (0.0) |  |
|  | PR | 11 (12.6) | 33 (21.0) |  |
|  | SD | 35 (40.2) | 61 (38.9) |  |
|  | PD | 33 (37.9) | 51 (32.5) |  |
|  | NE | 7 (8.0) | 12 (7.6) |  |
| Response |  | 12 (13.8) | 33 (21.0) | 0.173 |
| Disease control |  | 47 (54.0) | 94 (59.9) | 0.418 |

Only patients with target lesions were included in the analysis. ^a^Fisher’s exact test. *FP* fluoropyrimidine, *CR* complete response, *PR* partial response, *SD* stable disease, *PD* progressive disease, *NE* not evaluable
